# Improvements to the live-attenuated Newcastle disease virus vaccine using Carbopol^®^ 940 as a stabilizer

**DOI:** 10.14202/vetworld.2020.1641-1646

**Published:** 2020-08-19

**Authors:** Mahmoud Mohamed Abd El-Moneam, Nada Adel Fathy, Naglaa I. Ali, Heba Mohamed El Naggar

**Affiliations:** 1 Department of Newcastle Disease Vaccine Research, Veterinary Serum and Vaccine Research Institute, Agricultural Research Center, Cairo, Egypt; 2 Department of Pet Animal Vaccine Research, Veterinary Serum and Vaccine Research Institute, Agricultural Research Center, Cairo, Egypt; 3 Quality Control Laboratory, Veterinary Serum and Vaccine Research Institute, Agricultural Research Center, Cairo, Egypt

**Keywords:** Carbopol 940, LaSota, lyophilization, Newcastle disease virus, stabilizers, vaccine

## Abstract

**Background and Aim:**

One strategy that can be used to stabilize vaccines is to convert them into a dry powder. This can protect the integrity of the active ingredients as well as vaccine antigenicity during manufacture, storage, and transport. This study highlights the potent adjuvant activity of Carbopo^l®^ when used alone to stabilize live-attenuated Newcastle disease virus (NDV) vaccines or when used in a formulation together with skimmed milk. Tolerability and potency of these formulations were compared with those obtained from other local live NDV vaccines produced locally by the Veterinary Serum and Vaccine Research Institute.

**Materials and Methods:**

We evaluated the cellular and humoral immune responses to a locally prepared, live-attenuated LaSota virus vaccine. Vaccine formulations were stabilized with Carbopol^®^ 940 alone or in combination with skimmed milk.

**Results:**

Our results indicate that the use of Carbopol^®^ 940 alone to stabilize a live-attenuated LaSota vaccine resulted in enhanced cellular and humoral immunity. The antibody titer was prolonged through the 6^th^ week post-vaccination (5.0 log_2_). Full (100%) protection was observed in response to challenge with very virulent NDV at day 21 after vaccination; there were no clinical signs or lesions on examination. Addition of Carbopol^®^ 940 to the live-attenuated vaccine formulation resulted in a more compact, stable, and high-quality lyophilized cake after freeze-dried lyophilization compared with that produced by stabilization with skimmed milk alone.

**Conclusion:**

Our data suggest that Carbopol^®^ 940 may improve clinical responses to live-attenuated vaccines.

## Introduction

Newcastle disease (ND) has become one of the most important infections of poultry worldwide; infection is associated with respiratory, nervous system, enteric, and reproductive dysfunction. The causative agent of this disease is virulent ND virus (NDV), which belongs to the genus *Avulavirus* within the family *Paramyxoviridae* [1]. Live virus vaccines used to combat ND have been administered in drinking water, by aerosols or eye drops. Many virus infections are initiated at or through mucosal surfaces; as such, mucosal immunity may be the key to controlling initial infections with these pathogens. The most efficient mucosal immune responses are generated when vaccines are administered through this route; however, most vaccines are currently administered parenterally [2]. Generation of effective vaccines will require a combination of several approaches, including identification of suitable adjuvants that will present the antigen in a way that facilitates the induction of a sufficient and competent immune response with minimal to no adverse effects on recipients [3]. Furthermore, the adjuvant will need to be stable, cost-effective, and reliable from the pharmaceutical point of view; this would include a minimal cost per dose and low risk-to-safety ratio [4].

Carbomers are a class of cross-linked polyacrylic acids that are in wide use in biology and medicine, notably for topical applications and to facilitate drug delivery [5]. Carbomers have been evaluated as experimental adjuvants in veterinary vaccines against swine parvovirus [6], circovirus type 2 [7], *Staphylococcus aureus* in sheep [8], and equine influenza virus [9]. These reports revealed that biodegradable carbomers, such as Carbopol^®^, are safe for use in mammals and promote a more robust immune response than would be observed in response to antigen alone. Carbopol^®^ enhances cellular immunity by driving a strong type-1 T-cell (Th1) polarization and inducing production of interferon-gamma (IFNγ); it also promotes antigen capture by inflammatory macrophages. As such, Carbopol^®^ functions as a systemic adjuvant in animal models due to its capacity to promote strong pro-inflammatory Th1 polarization [5]. Despite its widespread use for veterinary applications, there is little information available in the published literature regarding the type and magnitude of the innate and/or adaptive immune responses induced by carbomers compared with that of other well-characterized adjuvants [10].

This study highlights the potent adjuvant activity of Carbopol^®^ when used alone to stabilize live-attenuated NDV vaccines or when used in a formulation together with skimmed milk. Tolerability and potency of these formulations were compared with those obtained from other local live NDV vaccines produced locally by the Veterinary Serum and Vaccine Research Institute (VSVRI).

## Materials and Methods

### Ethical approval

The study was approved by the Institutional Animal Ethics Committee of VSVRI. All procedures and the care of chicks were in accordance with the institutional guidelines for animal use in research.

### Study period and location

The study was conducted in Veterinary Serum and Vaccines Research Institute within the period from January 2019 to December 2019.

### Experimental chicks

One hundred and eighty chicks at 7 days of age were used to evaluate the test vaccines; 85 chicks at 5 days of age were used to evaluate safety. Specific pathogen-free (SPF) chicks were obtained from the SPF Egg Production Farm, Koum Oshein, El-Fayoum, Egypt.

### Viruses and stabilizers

The NDV (LaSota strain) was used for vaccine formulation; the very virulent NDV (vvNDV) field isolate (infectivity titer 10^6^ EID_50_/0.1 mL) [11] was used for the potency (challenge) test. Both viruses were obtained from the Poultry Viral Vaccine Department, VSVRI, Abbasia, Egypt.

Carbopol^®^ 940 NF polymer was supplied by Lubrizol^®^; it is a fluffy white powder composed of high-molecular-weight acrylic acid polymers cross-linked with allyl ethers of pentaerythritol. The Carbopol^®^ 940 NF polymer meets the criteria cited in the United States Pharmacopeia/National Formulary. The polymer was dissolved in hot double-distilled water with final concentration of 0.25%, then sterilized by autoclaving at 121°C for 20 min and stored at 4°C until further use. Before use, the Carbopol^®^ solutions were neutralized to pH 7.3 with 20% NaOH. Dried skim milk was from Dairy America^®^, Fresno, CA, USA; the dried powder was diluted to a concentration of 10% and autoclaved at 121°C for 20 min.

### Vaccine formulations

Three vaccine formulations were prepared and evaluated after immunization in SPF chicks, the inoculated dose was 1 mL per chick as described in Table-1.

**Table-1:** Vaccine formulations and animal experimental design.

Treatment with vaccines	Chicks/group	Age at immunization	Route
1. Group 1	50	7 days	Oral/drinking water
2. Group 2	50	7 days	Oral/drinking water
3. Group 3	50	7 days	Oral/drinking water
4. Group 4	30	7 days	Not vaccinated

Group 1: chicks vaccinated with lyophilized live attenuated NDV vaccine formulated with (Carbopol^®^ 940) alone as a stabilizer.

Group 2: chicks vaccinated with lyophilized live attenuated NDV vaccine formulated with a combination of (Carbopol^®^ 940) and skimmed milk as a stabilizer.

Group 3: chicks vaccinated with lyophilized live attenuated NDV vaccine formulated with skimmed milk as a stabilizer.

Group 4: unvaccinated chicks

One formulation of lyophilized live-attenuated NDV LaSota vaccine was prepared using Carbopol^®^ 940 NF alone (Group 1) with 3:2 virus to Carbapol^®^ 940. A second lyophilized live-attenuated NDV LaSota vaccine was prepared with Carbopol^®^ 940 NF together with skimmed milk (Group 2). A third formulation was produced locally by VSVRI and included lyophilized live-attenuated NDV LaSota vaccine prepared with skimmed milk alone (Group 3).

### Tests for sterility and safety

Standard sterility tests were applied to confirm that the prepared vaccines were free from bacterial, mycoplasma, and fungal contamination. These were performed by inoculation of the vaccine formulations into (1) tryptone soy broth followed by incubation at 25°C for 5 days, (2) thioglycollate broth followed by incubation at 37°C for 72 h, and (3) pleuropneumonia-like organisms broth incubated at 37°C for 15 days.

The vaccine preparations were tested for safety in 75 SPF chicks that were 5 days of age (25 chicks per vaccine formulation). Each chick was inoculated intraocularly with 10 doses of prepared live vaccine, and 10 chicks remained as controls; all chicks were observed for 21 days. As previously described, no chickens should show serious clinical signs and none should die from causes attributable to the vaccine [12]. Some chicks were subjected to postmortem examinations to detect any pathological lesions.

### Experimental design

#### Challenge experiment

The challenge experiment was performed using two different routes of virus administration, including both intramuscular (I/M) and intranasal (I/N) routes. Two groups of 15 vaccinated birds from Groups 1, 2, and 3 and 15 control birds were challenged through the I/M (trial 1) and I/N (trial 2) routes at 3 weeks post-vaccination with at least 10^4^ EID_50_ units of vvNDV [11]. Chicks were observed for 14 days, and the degree of protection was assessed according to the severity of the clinical signs and the mortality associated with virus challenge [12].

#### Cell proliferation assay

Heparinized blood samples were taken from chickens in all vaccinated and negative control groups at days 3, 7, 15, and 21 post-vaccination. Peripheral blood mononuclear cells were adjusted to 5-20 × 10^6^ cells/mL in phosphate-buffered saline. Proliferation was evaluated using a commercial cell proliferation kit II (XTT; Cat. No.11465015001, Sigma-Aldrich, St. Louis, MO, USA). Optical density (OD) was measured using an ELISA reader [13].

#### Serological responses

Serum samples were collected weekly through week 6 post-vaccination for the detection of serum antibodies by hemagglutination inhibition (HI) test. The sera were inactivated at 56°C for 30 min and stored at −20°C. Hemagglutination and HI tests were carried out following previously published protocols [12] using four hemagglutinating units of homologous antigen (NDV LaSota strain) to determine antibody titers in sera of vaccinated and unvaccinated chickens.

## Results

### Physical appearance (Figure-1)

**Figure-1:**
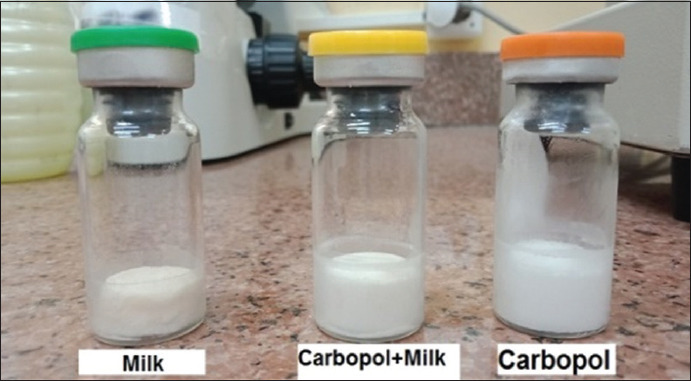
Physical appearance.

The lyophilized vaccine formulation prepared with skimmed milk alone as a stabilizer was yellowish, uniform in shape, and somewhat non-adherent to the vial walls; by contrast, the lyophilized vaccine formulations prepared with Carbopol^®^ 940 alone or in combination with skimmed milk were whitish uniform disks that remained situated within the walls of the vials.

### Cell-mediated immune response

**Figure-2:**
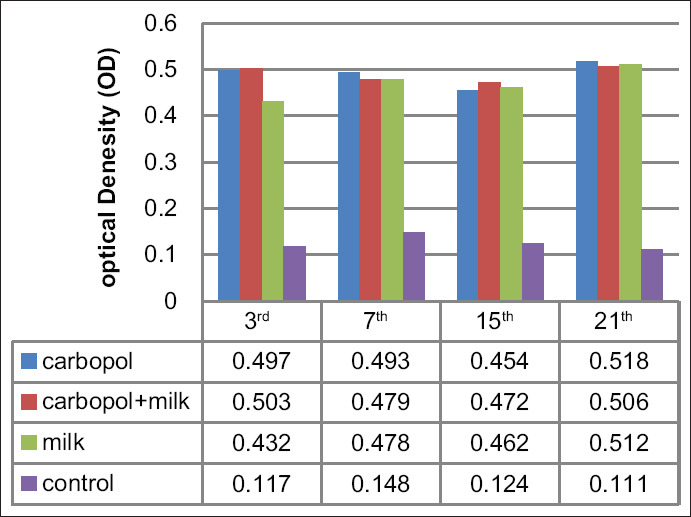
Cellular immune response results in chicks vaccinated by live Newcastle disease virus vaccines with different stabilizers.

The OD readings determined by ELISA indicated gradual increases in cell proliferation in all vaccinated groups. The highest level of cell proliferation was determined from blood samples at days 7 and 21 for all vaccinated groups except for Group 2 (stabilized with both Carbopol^®^ 940 and skimmed milk); the highest levels of cell proliferation in this latter group were detected on days 3 and 21 (Figure-2).

### Serological response

**Figure-3:**
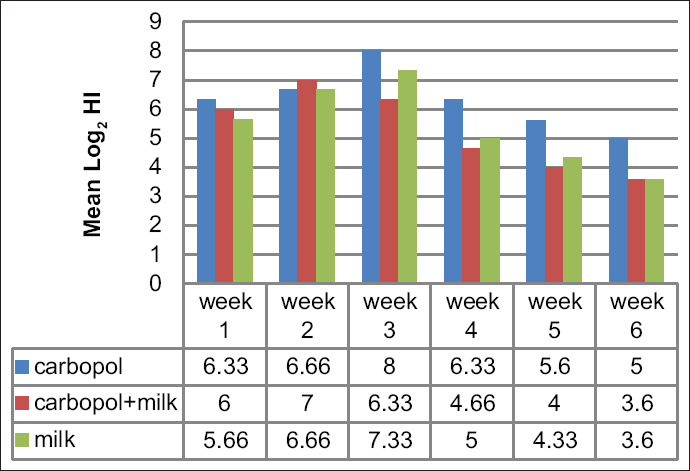
Mean Log_2_ HI (Newcastle disease [ND]) antibody titers in vaccinated chicken by live ND virus vaccine with different stabilizers.

Serological responses of vaccinated chickens were monitored by the HI test each week for a period of 6 weeks. All vaccinated groups exhibited high antibody titers. Highest antibody titers were observed among chickens in Group 1 (vaccines stabilized with Carbopol^®^ 940 alone); in this group, titers reached to 8 log_2_ at 21 days post-vaccination and remained the highest among these groups at 6 weeks ­post-vaccination. By contrast, low antibody titers were detected in all other vaccinated groups at 6 weeks post-vaccination (Figure-3).

### Vaccine potency

Satisfactory levels of protection were achieved in all groups vaccinated with live-attenuated NDV in response to challenge with vvNDV [11] (Tables-2 and 3).

**Table-2:** Potency of the prepared vaccines on SPF chicks against vvNDV at 21^th^ post-vaccination by I/M route (Trial 1).

Groups	No. of inoculated chicks	No. of dead/diseased birds	No. of survived birds	Protection%
1. Carbopol	15	0	15	100
2. Carbopol+milk	15	1	14	93
3. Milk	15	0	15	100
Control	15	15	0	0

NDV=Newcastle disease virus, SPF=Specific pathogen-free

**Table-3:** Potency of the prepared vaccines on SPF chicks against vvNDV at 21^th^ post-vaccination by Intra/Nasal route (Trial 2).

Groups	No. of inoculated chicks	No. of dead/diseased birds	No. of survived birds	Protection%
1. Carbopol	15	0	15	100
2. Carbopol+milk	15	0	15	100
3. Milk	15	0	15	100
Control	15	15	0	0

NDV=Newcastle disease virus, SPF=Specific pathogen-free

## Discussion

Ongoing discovery and development of adjuvants to be used in novel vaccine formulations may serve to increase safety and potency and/or may reduce the amount of antigen required to promote adaptive immune responses [10]. The use of polymers in ­vaccine formulations could result in improved antigen delivery and thus enhanced activation of appropriate immune responses without the need for multiple doses as typically employed in conventional vaccination strategies [3]. Polymers may also facilitate the controlled release of vaccine antigens over longer periods of time and may also serve as adjuvants for enhancing the immunogenicity of weaker antigens [14]. Carbopol^®^ is a polymeric formulation that has been widely used for this purpose; its capacity to form gels in aqueous solution facilitates its compatibility with many active ingredients [15]. Similarly, due to its low reactivity, its capacity to preserve virus antigens, and its efficacy when used in single-dose vaccination strategies, Carbopol^®^ is now in wide use as a vaccine adjuvant in the veterinary field [16].

In this study, we prepared three formulations of live NDV (LaSota) vaccines using different stabilizers. After lyophilization, live ND vaccine formulations prepared with Carbopol^®^ 940 were uniformly white and stable. After its neutralization to pH 7.3 with NaOH, the acidic Carbopol^®^ polymer solutions were able to absorb and retain water and to form irreversible agglomerates of polymer chains [17]. By contrast, the conventional live ND vaccine prepared from skimmed milk was uniformly yellowish-white and appears to have contracted away from the walls of their vials; this may be due to an abnormally high amount of water within this preparation that remains unfrozen water inside skimmed milk that evaporates during the drying stages, thus resulting in the observed volume contraction [18].

Testing revealed that all NDV vaccine preparations were free from bacterial and fungal contamination; furthermore, none of the vaccinated chicks experienced any local or systemic reactions or any mortality from the beginning through the end of the observation period. These results are in agreement with those previously described [19] and are consistent with information provided in the safety data sheet for Carbopol^®^ 940 and likewise with previous reports documenting no harm associated with carbomer preparations used in mammals [5].

There is relatively little information available regarding the type and magnitude of the adaptive immune response induced by Carbopol^®^ when compared with that of other well-characterized adjuvants [5]. Cell proliferation assays revealed gradual increases in all vaccinated groups. The highest level of activity was observed on days 7 and 21 in samples from Group 1 (Carbopol^®^ 940 alone) and Group 3 (skimmed milk alone); among samples from chickens in Group 2 (Carbopol^®^ 940 and skimmed milk), the highest level of activity was observed in days 3 and 21 post-vaccination (Figure-2). These results agreed with those published previously [20]; this previous study also provided evidence indicating that Carbopol^®^ functioned as an enhancer and modulator of immunity through its capacity to induce an early IFNγ response and by driving T-cell differentiation toward specific effector phenotypes. IFNs as a group are important members of host innate immunity and serve to prevent virus infection through direct antiviral effects and by triggering apoptosis [21]. The current data suggest that the impact of Carbopol^®^ on cell-mediated and humoral immunity was at its highest at day 21 post-vaccination, at the time of challenge with vvNDV. Notably, chickens in Group 1 (vaccinated with a formulation stabilized with Carbopol^®^ 940 alone) recorded an OD of 0.518 in the cell proliferation assay and (8 log_2_) for HI antibody titer and provided 100% protection against mortality associated with virulent virus challenge. The responses of chickens in Group 2 (vaccine prepared with Carbopol^®^ with skimmed milk) and Group 3 (vaccine prepared with skimmed milk alone) were slightly lower; ODs recorded from the cell proliferation assay were 0.506 and 0.512, respectively, and the HI assay titers were 6.33 log_2_ and 7.33 log_2_, respectively, at day 21 post-vaccination. Antibody titers against NDV detected by HI test revealed that the mean log_2_ serum neutralizing antibody titer among chickens in Group 1 began to increase beginning at the 1^st^ week ­post-vaccination (6.33 log_2_); titers reached their highest levels at week 3 post-vaccination (8.0 log_2_) and persisted as the highest among the three vaccinated groups through week 6 post-vaccination (5.0 log_2_). By contrast, HI titers determined for chickens in Groups 2 and 3 (skimmed milk) were lower than those detected in Group 1 at the 1^st^ week post-vaccination, at 6.0 log_2_ and 5.66 log_2_, respectively. At 3 weeks ­post-vaccination, slightly higher titers were detected, at 6.33 log_2_ and 7.33 log_2_ for Groups 2 and 3, respectively; antibody titer then underwent a gradual decrease until reaching its lowest level at week 6 post-vaccination (3.6 log_2_). These results agreed with those published previously [22] as part of a study that reported that the cross-linked Carbopol^®^ network results in antigen entrapment within the hydrogel domains. After hydration, the polymer chains may increase up to 10 times their original diameter; likewise, the ionization process leads to swelling of the cross-linked molecules, which then form a microgel network with stronger bonds [17]. As such, Carbopol^®^-based formulations may be optimal for controlled release of small molecules and may facilitate the slow release of antigen for a longer period of time; together with its capacity for immunomodulation through strong antigen presentation, this agent may be highly valuable when used to promote improved vaccination strategies [14,23].

The potency trials revealed that all vaccine formulations were associated with a high rate of protection against challenge with virulent virus. In trial 1 (challenged through the I/M route), chickens in Groups 1, 2, and 3 received 100%, 93%, and 100% protection against the sequelae of virulent infection, respectively (Table-2). This result was compatible with previously reported results [10]; results from this latter study suggest that the major immunomodulatory activity was associated with the carbomer component (i.e., Carbopol^®^) that was not harmful to mammals and stimulated a more robust immune response than that observed in response to antigen alone. In trial 2 (challenged through the I/N route), 100% protection was observed in all virus-challenged vaccinated groups versus 0% for the virus-challenged control group. This result is consistent with that reported previously [14] in a study that demonstrated the mucoadhesive properties of Carbopol^®^ associated with antigen trapping at the mucosal surface for longer periods of time. Our findings are also consistent with those of another study [20] that reported that the addition of Carbopol^®^ to modified live-attenuated virus vaccines improves cellular responses by early induction of IFNγ-producing cells.

## Conclusion

Our findings provide insight into a new formulation of live-attenuated LaSota vaccine that was developed using the stable, safe, and inexpensive stabilizer, Carbopol^®^ 940. Our results suggest that the addition of Carbopol^®^ 940 to a live-attenuated LaSota vaccine promotes enhancement and modulation of critical immune responses. As such, the use of Carbopol^®^ 940 as a stabilizer for live vaccines promotes health, safety, and economic benefits.

## Authors’ Contributions

NIA prepared the Carbopol^®^ 940. MMA, NAF, and HME conducted the laboratory animal experimental work drafted and revised the manuscript. All authors have read and approved the final manuscript.

## Competing Interests

The authors declare that they have no competing interests.

## Publisher’s Note

Veterinary World remains neutral with regard to jurisdictional claims in published institutional affiliation.

## References

[1] Maclachlan N.J, Dubovi E.D. (2016). Fenner's Veterinary Virology.

[2] Henderson A, Propst K, Kedlc R, Dow S. (2011). Mucosal immunization with liposome-nucleic acid adjuvant generates effective humoral and cellular immunity. Vaccine.

[3] Gartlana K.H, Krashiasa G, Wegmanna F, Hillsona W.R, Schererb E.M, Greenbergb P.D, Eisenbarthc S.C, Moghaddama A.E, Sattentauaa Q.J. (2016). Sterile inflammation induced by carbopol elicits robust adaptive immune responses in the absence of pathogen-associated molecular patterns. Vaccine.

[4] Kaurav M, Madan J, Sudheesh M.S, Pandey R.S. (2018). Combined adjuvant-delivery system for new generation vaccine antigens: Alliance has its own advantage. Artif. Cells Nanomed. Biotechnol.

[5] Gartlan K.H, Krashias G, Wegmann F, Hillson W.R, Scherer E.M, Greenberg P.D, Eisenbarth S.C, Moghaddam A.E, Sattentau Q.J. (2016). Sterile inflammation induced by Carbopol elicits robust adaptive immune responses in the absence of pathogen-associated molecular patterns. Vaccine.

[6] Gualandi G.L, Losio N.M, Muratori G, Foni E. (1988). The ability by different preparations of porcine parvovirus to enhance humoral immunity in swine and guinea pigs. Microbiologica.

[7] Hoogland M.J, Opriessnig T, Halbur P.G. (2006). Effects of adjuvants on porcine circovirus Type 2-associated lesions. J. Swine Health Prod.

[8] Tollersrud T, Nørstebø P.E, Engvik J.P, Andersen S.R, Reitan L.J, Lund A. (2002). Antibody responses in sheep vaccinated against *Staphylococcus aureus* mastitis: A comparison of two experimental vaccines containing different adjuvants. Vet. Res. Commun.

[9] Mumford J.A, Wilson H, Hannant D, Jessett D.M. (1994). Antigenicity and immunogenicity of equine influenza vaccines containing a carbomer adjuvant. Epidemiol. Infect.

[10] Wegmann F, Moghaddam A.E, Schiffner T, Gartlan K.H, Powell T.J, Russell R.A, Baart M, Carrow E.W, Sattentau Q.J. (2015). The carbomer-lecithin adjuvant adjuplex has potent immunoactivating properties and elicits protective adaptive immunity against influenza virus challenge in mice. Clin. Vaccine Immunol.

[11] Reda I.M, Sheble A. (1976). Cited by Khafagy A.K.

[12] OIE Terrestrial Manual (2019). Newcastle Disease.

[13] Scudiero D.A, Shoemaker R.H, Paull K.D, Monks A, Tierney S, Nofziger T.H, Currens M.J, Seniff D, Boyd M.R. (1988). Evaluation of soluble tetrazolium/Formazan Assay for cell growth and drug sensitivity in culture using human and other tumor cell lines. Cancer Res.

[14] Shakya A.K.N, Kumar K.S. (2012). Applications of polymeric adjuvants in studying autoimmune responses and vaccination against infectious diseases. J. R. Soc. Interface.

[15] Dinte E, Leucuta S.E. (2004). Farmacia LII.

[16] Diamantstein T, Wagner B, Beyse I, Odenwald M.V, Schulz G. (1971). Stimulation of humoral antibody formation by polyanions. I. The effect of polyacrylic acid on the primary immune response in mice immunized with sheep red blood cells. Eur. J. Immunol.

[17] Varges P.R, Costa C.M, Fonseca B.S, Naccache M.F, de Souza Mendes P.R. (2019). Rheological characterization of carbopol^®^dispersions in water and in water/glycerol solutions. Fluids.

[18] Patel S.M, Nail S.L, Pikal M.J, Geidobler R, Winter G, Hawe A, Davagnino J, Gupta S.R. (2017). Lyophilized drug product cake appearance:What is acceptable?. J. Pharm. Prod.

[19] Code of American Federal Regulation-13CFR (2019). Office of the Federal Register National Archives Records Service. Animals and Animal Products.

[20] Mair K.H, Koinig H, Gerner W, Höhne A, Bretthauer J, Kroll J.J, Roof M.B, Saalmüller A, Stadler K, Libanova R. (2015). Carbopol improves the early cellular immune responses induced by the modified-life vaccine Ingelvac PRRS1 MLV. Vet. Microbiol.

[21] Hu Z, Hu J, Hu S, Liu X, Wang X, Zhu J, Liu X. (2012). Strong innate immune response and cell death in chicken splenocytes infected with genotype VIId Newcastle disease virus. Virol. J.

[22] Singla A.K, Chawla M, Singh A. (2000). Potential applications of carbomer in oral mucoadhesive controlled drug delivery system:A review. Drug Dev. Ind. Pharm.

[23] Rice-Ficht A.C, Arenas-Gamboa A.M, Kahl-McDonagh M.M, Ficht T.A. (2009). Polymeric particles in vaccine delivery. Curr. Opin. Microbiol.

